# Untargeted Metabolomics Reveals Dysregulation of Glycine- and Serine-Coupled Metabolic Pathways in an ALDH1L1-Dependent Manner In Vivo

**DOI:** 10.3390/metabo14120696

**Published:** 2024-12-10

**Authors:** Grace Fu, Sabrina Molina, Sergey A. Krupenko, Susan Sumner, Blake R. Rushing

**Affiliations:** 1 Department of Nutrition, University of North Carolina at Chapel Hill, Chapel Hill, NC 27599, USA; 2 Nutrition Research Institute, University of North Carolina at Chapel Hill, Kannapolis, NC 28081, USA

**Keywords:** ALDH1L1, folate, metabolomics, liver, plasma

## Abstract

**Background:** ALDH1L1 plays a crucial role in folate metabolism, regulating the flow of one-carbon groups through the conversion of 10-formyltetrahydrofolate to tetrahydrofolate and CO_2_ in a NADP^+^-dependent reaction. The downregulation of ALDH1L1 promotes malignant tumor growth, and silencing of ALDH1L1 is commonly observed in many cancers. In a previous study, *Aldh1l1* knockout (KO) mice were found to have an altered liver metabotype, including significant alterations in glycine and serine. Serine and glycine play crucial roles in pathways linked to cancer initiation and progression, including one-carbon metabolism. **Objective/Methods:** To further investigate the metabolic role of ALDH1L1, an untargeted metabolomic analysis was conducted on the liver and plasma of both KO and wild-type (WT) male and female mice. Since ALDH1L1 affects glycine- and serine-coupled metabolites and metabolic pathways, correlation analyses between liver glycine and serine with other liver or plasma metabolites were performed for both WT and KO mice. Significantly correlated metabolites were input into MetaboAnalyst 5.0 for pathway analysis to uncover metabolic pathways coupled with serine and glycine in the presence or absence of ALDH1L1 expression. **Results:** This analysis showed substantial alterations in pathways associated with glycine and serine following ALDH1L1 loss, including the amino acid metabolism, antioxidant pathways, fatty acid oxidation, and vitamin B5 metabolism. These results indicate the glycine- and serine-linked metabolic reprogramming following ALDH1L1 loss to support macromolecule biosynthesis and antioxidant defense. Additional research is required to further explore the correlation between specific alterations in these pathways and tumor growth, as well as to identify potential dietary interventions to mitigate the detrimental effects of ALDH1L1 loss.

## 1. Introduction

Folate coenzymes play a central role in one-carbon transfer reactions, thereby regulating amino acid biogenesis, nucleotide synthesis, and methylation reactions within the cell [1]. Cytosolic 10-formyltetrahydrofolate (10-CHO-THF) dehydrogenase (ALDH1L1) is a major enzyme in folate pathways that is found in particularly high levels in the liver and pancreas. ALDH1L1 converts 10-CHO-THF to THF and CO_2_ thus diverting one-carbon groups (OCG) from biosynthetic reactions. Previous studies have demonstrated that ALDH1L1 knockout mice exhibit altered metabolite profiles, notably altered serine and glycine levels, as well as functional folate deficiency, even when fed a folate-adequate diet, indicating the importance of ALDH1L1 in maintaining serine and glycine homeostasis and folate status [2]. The role of ALDH1L1 in regulating glycine and serine was also observed by knocking out this enzyme in RT4 cells [2]. ALDH1L1 is frequently and ubiquitously downregulated in human cancers due to promoter methylation [3]. Studies examining gene expression profiles have revealed ALDH1L1 as one of the most under-expressed genes in hepatocellular carcinomas (HCCs) as compared to normal livers [4]. Moreover, ALDH1L1 was strongly downregulated in metastatic liver carcinomas and late-stage or high-grade HCCs. Diminished expression of ALDH1L1 has been linked to poor prognosis in HCC, highlighting its critical role in cancer development and progression [5,6]. Also, re-expression of ALDH1L1 in cancers cells has been shown to reduce proliferation and induce apoptosis, suggesting that ALDH1L1 may be a putative tumor suppressor [7,8]. Given the pivotal role of ALDH1L1 in cancer, it is crucial to delve into its relationship with specific metabolic pathways, particularly those related to serine and glycine, to understand the enzyme’s impact on cellular function.

Serine and glycine, common proteogenic amino acids, play crucial roles in various cellular functions. Serine is involved in protein synthesis, neurotransmission, folate and methionine cycles, sphingolipid and phospholipid synthesis, and has neuroprotective effects in brain health [9,10]. It is also used in the treatment of neurological conditions like epilepsy and Alzheimer’s disease [10]. Glycine serves as a precursor for numerous key metabolites, including creatine, glutathione, purines, and porphyrins [11,12]. Glycine plays an important role in metabolic regulation, oxidative reactions, and neurological function, making it valuable in preventing diseases and supporting overall health [13]. Moreover, serine/glycine metabolism has been found to support tumor homeostasis and drives oncogenesis [14]. As shown in previous studies, the loss of ALDH1L1 leads to a decrease in THF, which reduces the generation of glycine from serine, highlighting the intricate connection between ALDH1L1 and amino acid metabolism [15].

To better understand the connection between ALDH1L1 and glycine/serine metabolism, we re-analyzed previous metabolomics data generated from wild-type and ALDH1L1 knockout mice which showed that this enzyme significantly decreased glycine and glycine conjugates while increasing serine [16]. Because these amino acids are critical components of folate metabolism, the goal of the current study was to further investigate how alterations in these amino acids during folate pathway disruption are linked to other metabolic processes, and how this could potentially inform of ALDH1L1’s tumor-suppressive effects. Using correlation analyses, we assessed metabolites and metabolic pathways coupled to serine and glycine under a normal folate status, and whether they become decoupled due to genetic disruption of folate metabolism. We also present this correlation analysis with the serine-to-glycine ratio, which has been shown previously to be more closely related to ALDH1L1 activity than either metabolite alone [17]. This information will provide new insights into folate metabolism that are distinct from the original study as it uses a novel approach that is central to serine and glycine to determine how these amino acids are linked to other metabolic pathways, and how this becomes dysregulated during folate pathway disruption. This information will lead to a better understanding of how ALDH1L1 regulates cellular metabolism by altering the functions of serine and glycine. This will aid in understanding the impact and relations of ALDH1L1 to cancer and other diseases linked to this enzyme.

## 2. Materials and Methods

### 2.1. Metabolomics Data

This study is a re-analysis of a previous metabolomics study involving *Aldh1l1* knockout mice that used typical univariate and multivariate analysis approaches, and full method details can be found in this previous publication [16]. Briefly, *Aldh1l1* knockout mice were generated using a previously reported method and were further back-crossed (8–10 generations) with C57Bl/6NHsd mice. Heterozygous males and females were intercrossed to obtain knockout (KO) and wild-type (WT) littermates, and plasma and liver were harvested for metabolomics.

A volume of 50 μL of plasma from each mouse was placed in pre-labeled 2.0 mL Lo-Bind Eppendorf tubes. Method blanks were prepared by adding 50 μL of LC–MS grade water to pre-labeled 2.0 mL Lo-Bind Eppendorf tubes and processed similarly to the study samples. A solution of 80% methanol (with 500 ng/mL Tryptophan-d5) was added at a volume of 400 μL to each tube. Samples were vortexed for 2 min at 5000 rpm, incubated at 4 °C for 10 min, and then centrifuged at 4 °C and 16,000× *g* for 10 min to pellet protein. Then, 350 μL of the supernatant was transferred to new tubes and dried overnight via SpeedVac. Samples were reconstituted with 100 μL of reconstitution solution (95:5 H_2_O: methanol), vortexed for 10 min at 5000 rpm, and centrifuged at 4 °C and 16,000× *g* for 10 min. Supernatants were then transferred to HPLC autosampler vials, and a total quality control study pool (QCSP) was prepared by combining 5 μL of each study sample. 5 μL were injected onto the LC–MS column for untargeted analysis.

Liver samples, pre-weighed (120–150 mg), were placed in MagNA Lyser tubes with ceramic beads. Cold homogenization solution (5 μL/mg of tissue; 80:20 methanol) was added while keeping the samples frozen. Method blanks were prepared by adding 500 μL of homogenization solution to empty MagNA Lyser tubes with beads, processed similarly to the study samples. Samples were homogenized at 5 m/s for 30 s using an Omni Bead Ruptor Elite. Protein and tissue debris were pelleted by centrifugation at 4 °C and 16,000× *g* for 10 min. Next, 200 μL of the supernatant was transferred to new pre-labeled 2.0 mL Lo-Bind Eppendorf tubes and dried overnight by SpeedVac. Samples were reconstituted with 500 μL of Tissue Reconstitution Solution (95:5 H_2_O with 500 ng/mL Tryptophan-d5), vortexed for 10 min at 5000 rpm, and centrifuged at 4 °C and 16,000× *g* for 10 min. Supernatants were transferred to autosampler vials, and 5 μL of each study sample was combined to create a total QCSP. A volume of 5 μL was injected for untargeted LC-MS analysis.

Metabolomics data were acquired using a Vanquish UHPLC system coupled with a Q Exactive™ HF-X Hybrid Quadrupole-Orbitrap Mass Spectrometer (Thermo Fisher Scientific, San Jose, CA, USA). Plasma or liver extract samples were randomized, with blanks and QCSPs inserted after every 10 study samples. Metabolites were separated on an HSS T3 C18 column (2.1 × 100 mm, 1.7 µm, Waters Corporation, Milford, MA, USA) at 50 °C, using a binary mobile phase of water (A) and methanol (B), each containing 0.1% formic acid (*v*/*v*). The UHPLC linear gradient started at 2% B, increased to 100% B in 16 min, and was held for 4 min at a flow rate of 400 μL/min. Untargeted data were acquired from 70 to 1050 m/z using a data-dependent acquisition mode. Progenesis QI (version 2.1, Waters Corporation) was used for peak picking, alignment, and normalization. Relative quantitation values were obtained by integrating peak areas for all metabolomic features. Background signals were filtered out by analyzing peaks with a mean peak abundance fold change > 3 in the total QCSP vs. blanks. Peaks were then normalized using the “normalize to all” feature in Progenesis QI. Plasma data signals with an RSD > 30% across total QCSPs were removed. Two liver samples were excluded from the analysis due to poor chromatogram shape/alignment.

Peak identification or annotation was performed through Progenesis QI by matching data to an in-house physical standards library (approximately 2000 compounds) and publicly available databases (HMDB, NIST, and METLIN). Matches were based on exact mass (MS), MS/MS fragmentation pattern, isotopic ion pattern, or retention time (RT, for in-house library standards). An ontology system developed by our lab denoted evidence for identification or annotation: OL1 (in-house library match by MS, MS/MS, and RT); OL2a (in-house library match by MS and RT); OL2b (in-house library match by MS and MS/MS); PDa (public database match by MS and experimental MS/MS); PDb (public database match by MS and theoretical MS/MS); PDc (public database match by MS and isotopic similarity); and PDd (public database match by MS only). In the current study, only metabolites that matched to the in-house library were used for analysis, which consists largely of compounds involved in primary metabolism including pathways related to the metabolism of amino acids, carboxylic acids, biogenic amines, nucleotides, nucleosides, acylcarnitines, mono- and disaccharides, saturated and unsaturated fatty acids, steroids, bile acids, hormones, and vitamins, which has been utilized in our previous studies [2,16,18,19,20]. Of note, metabolomics platforms cannot always distinguish between isomers, and the provided metabolite name corresponds to the purchased reference standard that is in the in-house library.

### 2.2. Correlation Analysis

To identify metabolites that significantly correlated with glycine, serine, or the serine-to-glycine ratio, a correlation analysis was performed by using the ”Statistical Analysis” module in MetaboAnalyst 5.0. All metabolomics data were input in the form of normalized peak intensities and correlation analyses were performed separately for WT (*n* = 15) and KO (*n* = 17). Serine-to-glycine ratios were calculated for each sample by dividing the peak intensity of serine by the peak intensity of glycine. Spearman rank correlation was used for the distance measure, and tables of correlation values and *p*-values of metabolites with glycine, serine, or the serine-to-glycine ratio were generated. Analyses were performed separately for liver and plasma samples.

### 2.3. Pathway Analysis

**Figure 1:**
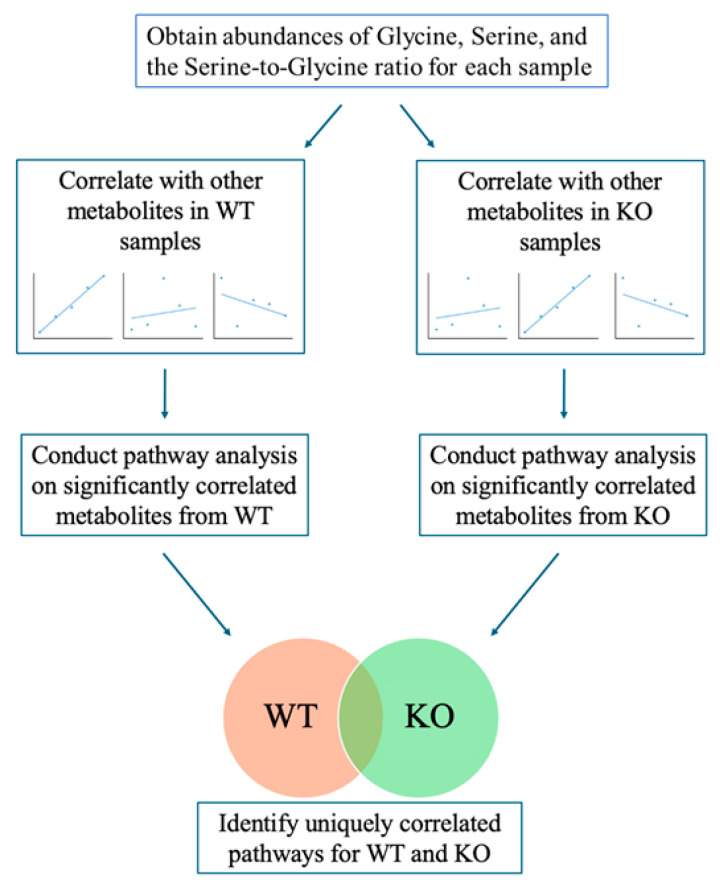
**Schematic of analytic approach.** Hepatic serine and glycine peak intensities and the ratio of serine-to-glycine peak intensities, were correlated with the intensities of other metabolites in the liver and plasma datasets for both WT and KO mice. Significantly correlated metabolites were then used as inputs for pathway analysis in MetaboAnalyst to determine metabolic pathways significantly correlated with serine, glycine, or the serine-to-glycine ratio for both liver and plasma, and results were compared between WT and KO samples.

A pathway analysis was also performed by using the MS Peaks to Pathways module in MetaboAnalyst. Metabolites that had a correlation *p*-value *<* 0.10 were selected for input compounds. Pathway analysis was performed separately for correlation results of WT and KO mice. A value of 0.10 was used for the *p*-value cutoff, and the Mus musculus (mouse) (KEGG) pathway library was used. Analyses were performed separately for liver and plasma samples (Figure 1). All reported pathway *p*-values were calculated using the Fisher’s exact test, and a *p*-value less than 0.1 was considered significant based on the sample size.

## 3. Results

### 3.1. Correlation of Serine and Glycine with Other Metabolites

**Figure 2:**
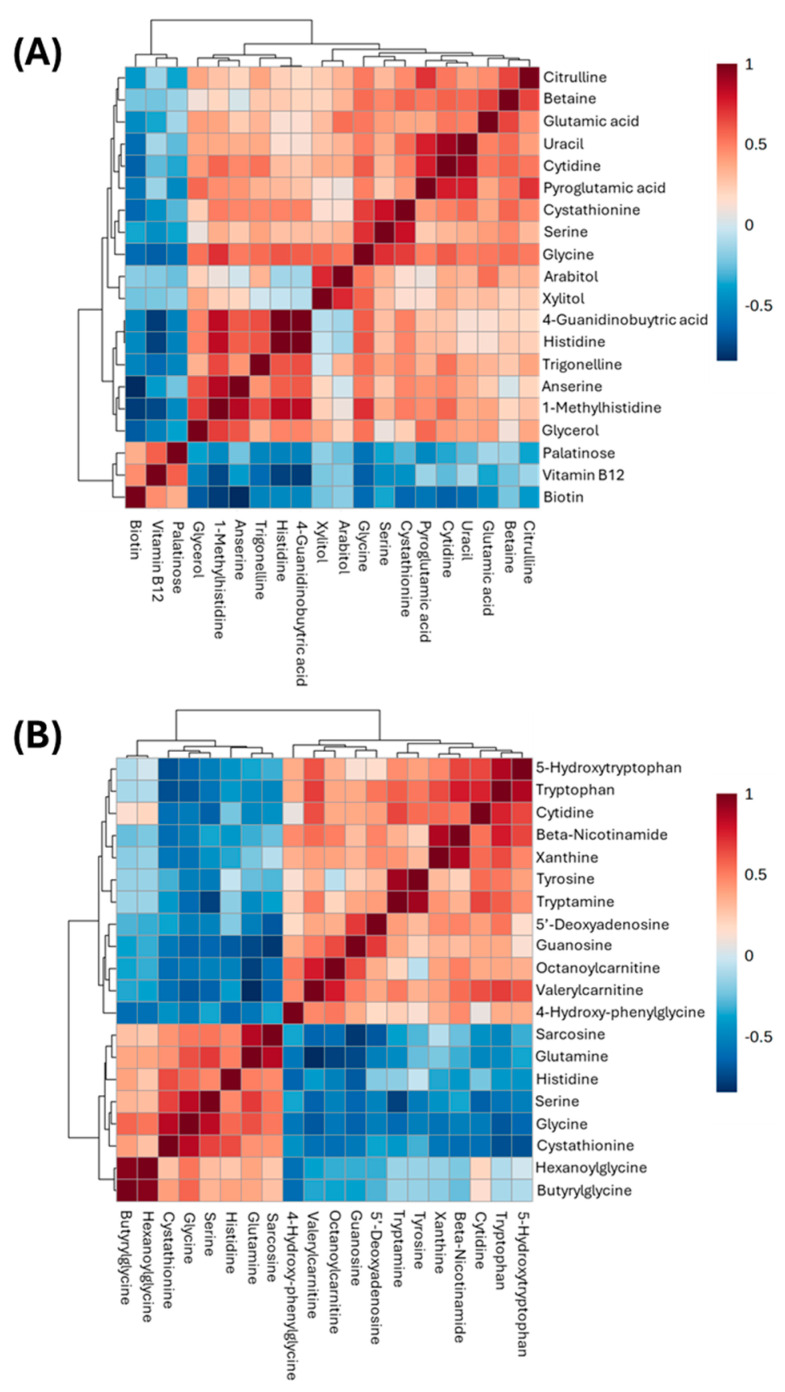
**Correlation heat map of liver and plasma metabolites using peak intensities in WT (A) and KO (B) mice.** Values within the heatmap are Spearman rank correlation values. Red indicates a strong positive correlation whereas blue indicates a strong negative correlation. For each heatmap, the top 20 correlated metabolites with glycine are displayed.

The correlation heat map in Figure 2 represents the entire metabolomic dataset in KO mice showing a strong negative (dark blue) and positive (dark red) correlation between each metabolite. This heatmap was built for both KO and WT mice separately to understand differences in metabolite correlations. The full list of metabolite correlation values and *p*-values is viewable in the Supplementary Material (Table S1). Table 1 is used as an example to show what liver metabolites are strongly positively or negatively correlated with glycine in the KO mice with *p*-value < 0.05. The top five metabolites are cystathionine, serine, tryptophan, valerylcarnitine, and guanosine, with *p*-values ranging from 1.71 × 10^−5^ to 5.44 × 10^−3^.

**Figure 3:**
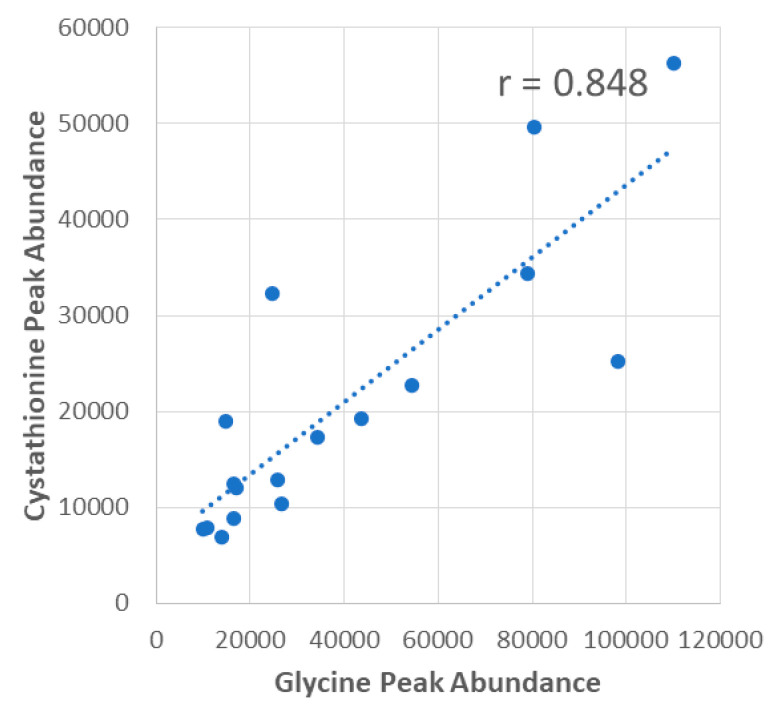
**Example correlation between liver glycine and liver cystathionine in ALDH1L1 KO mice.** Correlations were performed using peak area values that were calculated by Progenesis QI.

The scatterplot shown in Figure 3 shows an example of a statistically significant positive correlation between glycine and liver cystathionine peak abundances in the ALDH1L1 KO mice with a correlation coefficient (r) of 0.848. All liver metabolites with *p* < 0.10 were input into MetaboAnalyst’s Pathway Analysis module to determine metabolic pathways significantly correlated with glycine, serine, and the serine-to-glycine ratio in WT or KO mice. The same process was carried out for plasma metabolites, and a summary of pathways and their corresponding *p*-values for all comparisons is listed in Table 2.

### 3.2. Pathway Correlations with Glycine Using Liver Metabolites

**Figure 4:**
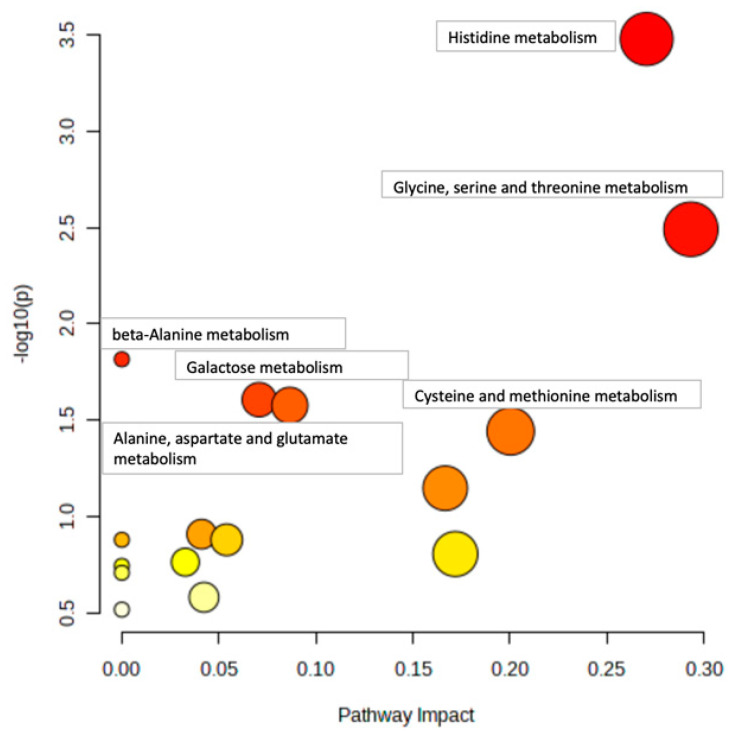
**Pathway analysis of liver metabolites correlated with glycine in WT mice.** Pathways with *p* < 0.05 are annotated.

**Figure 5:**
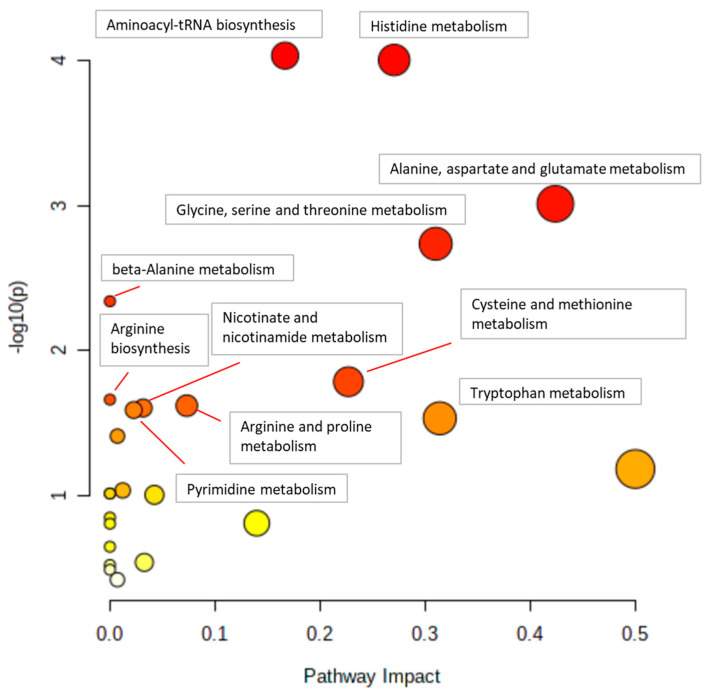
**Pathway analysis of liver metabolites correlated with glycine in KO mice.** Pathways with *p* < 0.05 are annotated.

Figure 4 and Figure 5 show example pathway analysis plots after using metabolites significantly correlated with glycine (*p* < 0.1) in WT (Figure 4) and KO (Figure 5) mice as inputs. For WT mice, the top five pathways correlated with glycine were histidine metabolism; glycine, serine, and threonine metabolism; beta-alanine metabolism; galactose metabolism; and alanine, aspartate, and glutamate metabolism, with *p*-values ranging from 3.34 × 10^−4^ to 2.64 × 10^−2^. For KO mice, the top five pathways most significantly correlated with glycine were aminoacyl-tRNA biosynthesis; histidine metabolism; alanine, aspartate, and glutamate metabolism; glycine, serine, and threonine metabolism; and beta-alanine metabolism, with *p*-values ranging from 1.08 × 10^−4^ to 4.95 × 10^−3^. Notably, galactose metabolism was only significant in WT mice, whereas arginine biosynthesis; arginine and proline metabolism; nicotinate and nicotinamide metabolism; pyrimidine metabolism; tryptophan metabolism; pantothenate and CoA biosynthesis; phenylalanine, tyrosine, and tryptophan biosynthesis; D-glutamine and D-glutamate metabolism; and nitrogen metabolism were the pathways that were only significantly correlated to glycine in KO mice.

### 3.3. Pathway Correlations with Serine Using Liver Metabolites

The top five pathways most significantly correlated to serine in WT mice were nicotinate and nicotinamide metabolism; glycine, serine and threonine metabolism; arginine biosynthesis; histidine metabolism; and purine metabolism with *p*-values ranging from 3.39 × 10^−4^ to 2.51 × 10^−2^. For KO mice, the top five pathways that were most significantly correlated with serine were aminoacyl-tRNA biosynthesis; histidine metabolism; arginine biosynthesis; phenylalanine, tyrosine, and tryptophan biosynthesis; and beta-alanine metabolism, with *p*-values ranging from 1.36 × 10^−7^ to 7.45 × 10^−3^. Glutathione metabolism and linoleic acid metabolism were pathways only significantly correlated to serine in WT mice. Phenylalanine, tyrosine, and tryptophan biosynthesis; phenylalanine, tyrosine, and tryptophan biosynthesis; tryptophan metabolism; phenylalanine metabolism; cysteine and methionine metabolism; pyrimidine metabolism; and pantothenate and CoA biosynthesis were the pathways that were only significantly correlated to serine in KO mice.

### 3.4. Pathway Correlations with the Serine-to-Glycine Ratio Using Liver Metabolites

The pathways that most significantly correlated with the serine-to-glycine ratio in WT mice were beta-alanine metabolism; glutathione metabolism; histidine metabolism; lysine degradation; and linoleic acid metabolism, with *p*-values ranging from 2.22 × 10^−4^ to 7.52 × 10^−2^. The top pathways that most significantly correlated with the serine-to-glycine ratio in KO mice were glutathione metabolism; glycine, serine, and threonine metabolism; arginine and proline metabolism; beta-alanine metabolism; and galactose metabolism, with *p*-values ranging from 9.66 × 10^−6^ to 3.77 × 10^−2^. Lysine degradation and purine metabolism were the pathways that were only significantly correlated to the serine-to-glycine ratio in WT mice. Galactose metabolism; arginine and proline metabolism; and glycerophospholipid metabolism were the pathways that were only significantly correlated to the serine-to-glycine ratio in KO mice.

### 3.5. Pathway Correlations with Glycine Using Plasma Metabolites

The top five pathways that most significantly correlated with glycine in WT mice were arginine biosynthesis; glutathione metabolism; pyrimidine metabolism; nitrogen metabolism; and D-glutamine and D-glutamate metabolism, with *p*-values ranging from 2.78 × 10^−3^ to 3.54 × 10^−2^. Nitrogen metabolism as well as D-glutamine and D-glutamate metabolism have the same *p*-value of 3.54 × 10^−2^. These pathways were only significant in WT mice compared to KO mice. Pathways that significantly correlated with glycine in KO mice were vitamin B6 metabolism; lysine degradation; cysteine and methionine metabolism; and arginine and proline metabolism, with *p*-values ranging from 2.39 × 10^−2^ to 8.76×10^−2^. These pathways were only significant in KO mice compared to WT mice.

### 3.6. Pathway Correlations with Serine Using Plasma Metabolites

The most significant pathways correlated with serine in WT mice were tryptophan metabolism; aminoacyl-tRNA biosynthesis; riboflavin metabolism; nicotinate and nicotinamide metabolism; and histidine metabolism, with *p*-values ranging from 1.01 × 10^−2^ to 6.22 × 10^−2^. Riboflavin metabolism; nicotinate and nicotinamide metabolism; histidine metabolism; and beta-alanine metabolism were only significantly correlated to serine in WT mice compared to KO mice. The top five pathways that were significantly correlated with serine in KO mice were tryptophan metabolism; pantothenate and CoA biosynthesis; aminoacyl-tRNA biosynthesis; valine, leucine, and isoleucine biosynthesis; and biotin metabolism, with *p*-values ranging from 2.84 × 10^−4^ to 8.33 × 10^−2^. Pantothenate and CoA biosynthesis; aminoacyl-tRNA biosynthesis; valine, leucine, and isoleucine biosynthesis; and biotin metabolism were only significantly correlated to serine in KO mice.

### 3.7. Pathway Correlations with the Serine-to-Glycine Ratio Using Plasma Metabolites

The top five pathways that were significantly correlated with the serine-to-glycine ratio in WT mice were glycine, serine, and threonine metabolism; tryptophan metabolism; histidine metabolism; aminoacyl-tRNA biosynthesis; and sphingolipid metabolism, with *p*-values ranging from 9.01 × 10^−3^ to 3.15 × 10^−2^. Glycine, serine, and threonine metabolism; tryptophan metabolism; histidine metabolism; sphingolipid metabolism; lysine degradation; and arginine and proline metabolism were only significantly correlated to the serine-to-glycine ratio in WT mice. The top pathways that were significantly correlated with the serine-to-glycine ratio in KO mice were aminoacyl-tRNA biosynthesis; purine metabolism; nitrogen metabolism; D-glutamine and D-glutamate metabolism; and alanine, aspartate, and glutamate metabolism, with *p*-values ranging from 1.94 × 10^−4^ to 4.22 × 10^−3^. Purine metabolism; nitrogen metabolism; D-glutamine and D-glutamate metabolism; alanine, aspartate, and glutamate metabolism; arginine biosynthesis; glutathione metabolism; phenylalanine, tyrosine, and tryptophan biosynthesis; glyoxylate and dicarboxylate metabolism; and tyrosine metabolism were only significantly correlated to the serine-to-glycine ratio in KO mice.

## 4. Discussion

**Figure 6:**
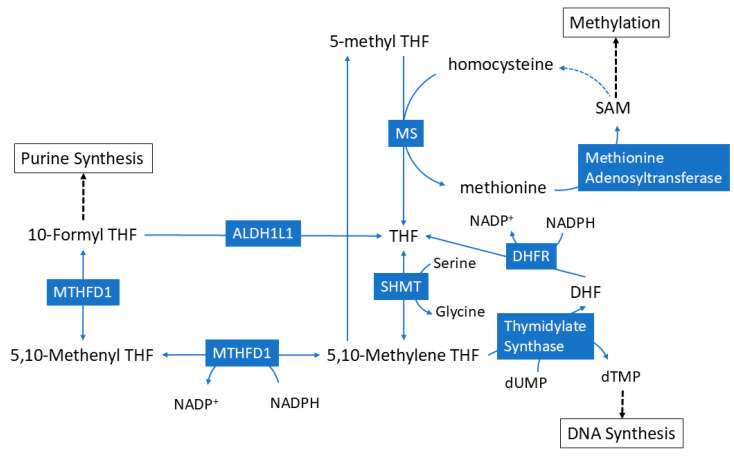
**Schematic showing the role of ALDH1L1 in folate metabolism**.

The goal of the current study was to assess metabolites and metabolic pathways that were significantly coupled with hepatic serine and glycine, and to identify how ALDH1L1 status affects this coupling. This information would provide better insight into the fate of serine and glycine during the loss of ALDH1L1 and bring a better understanding of how the loss of this enzyme contributes to tumorigenesis. As shown in Figure 6, ALDH1L1 regulates the conversion of serine to glycine, and these two amino acids have been shown to play key roles in oncogenesis, including the regulation of cancer cell proliferation [21,22]. Here, we employed a unique approach that takes advantage of a large metabolomics dataset where hepatic glycine and serine levels were correlated with other metabolites in order to identify metabolites whose intensities are coupled with these two amino acids. Significantly correlated metabolites were then subjected to pathway analysis to uncover metabolic pathways that were significantly correlated with either glycine or serine. Notably, we used Spearman’s correlations because this approach does not require a normal distribution of the data and has also been shown to be superior compared to other correlation methods for omics data [23]. In WT mice, we observed several hepatic metabolites within one-carbon pathways that significantly correlated with hepatic glycine, serine, or both. In general, fewer plasma metabolites significantly correlated with serine or glycine as compared to hepatic metabolites. Loss of ALDH1L1 generally led to more metabolites that were significantly correlated with serine or glycine, which corresponded to a larger number of significant pathways. This is consistent with previous findings that loss of ALDH1L1 leads to widespread changes in the metabolome [1,2,15,16]. The data from the current study provide more insight into how the loss of ALDH1L1 changes the metabolic role of serine and glycine. An examination of these pathways has the potential to unveil the pivotal roles of these amino acids during this state of metabolic stress, and their implications in cancer formation following ALDH1L1 loss. These identified pathways may offer insights into potential avenues for clinical and dietary interventions in individuals with compromised ALDH1L1 activity (Figure 6).

**Figure 7:**
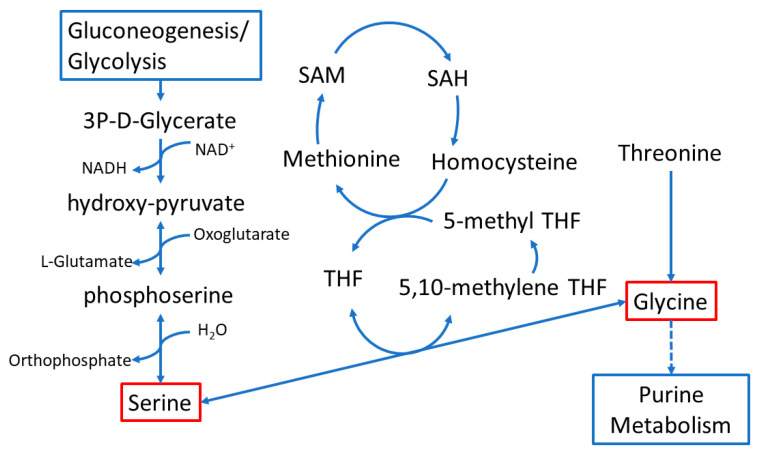
**Schematic depicting the interaction of folate with glycine, serine, and threonine metabolism**.

Glycine and serine are intricately connected in the metabolic pathway illustrated in Figure 7 [14]. This pathway involves various molecular precursors, including glycerate from gluconeogenesis or glycolysis, cysteine and methionine metabolism, and purine metabolism. Additionally, glycine is used in the synthesis of bile acids that are necessary for the digestion and absorption of dietary fats. While they can be produced in humans, de novo synthesis of serine and glycine can be insufficient under certain conditions, making them conditionally essential amino acids [9,13]. Because of their connection with pathways that control cellular energetics, antioxidant production, and nucleic acid synthesis, serine and glycine play a significant role in controlling macromolecule synthesis, cellular proliferation, and antioxidant defense. This is particularly relevant during tumorigenesis where these processes become critical for cancer cell survival and progression [14].

Following the knockout of ALDH1L1, many additional metabolic pathways became significantly associated with serine and glycine in the liver. The majority of these pathways are related to amino acid metabolism including conversions of arginine, proline, beta-alanine, phenylalanine, tyrosine, and tryptophan. Additionally, aminoacyl-tRNA biosynthesis was only marginally significant in WT mice, but was strongly significant in KO mice. Taken together, these data suggest that glycine and serine levels become more coupled to amino acid metabolic pathways following ALDH1L1 loss. ALDH1L1 regulates the flux of one-carbon groups from the folate pool to anabolic reactions thus providing a mechanism to control cellular proliferation. In support of this function, loss of ALDH1L1 has been shown to accelerate the proliferation of cancer cells. In agreement with this finding, the enzyme is also downregulated in mouse fibroblasts during the S-phase of the cell cycle [24]. During the active proliferation state, cells must rewire their metabolism to support biomass accumulation. Amino acid acquisition is a critical part of this process, and cells can rewire the metabolism to facilitate the uptake of amino acids from the extracellular environment or redirect intracellular metabolic pathways for amino acid biosynthesis [25]. In the original study, we identified several amino acid pathways which were significantly altered due to ALDH1L1 loss. Our data suggest that following ALDH1L1 loss, serine and glycine become more coupled with other amino acid pathways, suggesting that these two amino acids become more essential toward protein synthesis or other anabolic processes.

Interestingly, we found that glutathione synthesis was significantly correlated with serine in WT mice, but not in KO mice. This suggests that during ALDH1L1 loss, serine is more heavily shuttled towards other amino acid pathways instead of glutathione synthesis. This may impair the ability of *Aldh1l1^-/-^* cells to respond to oxidative stress compared to WT cells. Previous studies have shown that the knockout of ALDH1L1 reduced the antioxidant pool of mouse livers and caused an overall elevation of pro-inflammatory genes [15]. A decreased flux of serine towards glutathione production and therefore a lower antioxidant capacity would be in agreement with these results. Serine and glycine participating more heavily in anabolic processes (protein synthesis) and decreasing the synthesis of glutathione and other antioxidants may create a cellular environment that is more favorable towards tumorigenesis and other pathologies related to uncontrolled proliferation and/or oxidative damage.

Interestingly, many of the strongest correlations in this study involved acylcarnitines and the serine-to-glycine ratio. Hepatic levels of carnitine and all 11 measured acylcarnitines were significantly positively correlated with the serine-to-glycine ratio in WT mice and had an r > 0.5, while only one acylcarnitine was significantly correlated in the KO mice (Table S1). This overall pattern was also seen when correlating the serine-to-glycine ratio to plasma metabolites, although the KO mice had more significant correlations, albeit in the opposite direction. As acylcarnitines are intermediates in the mitochondrial beta-oxidation of fatty acids, this suggests that the conversion of serine to glycine is coupled with the mitochondrial metabolism of fatty acids and that ALDH1L1 is critical for this coupling. Previous studies have suggested a role for ALDH1L1 in regulating mitochondrial metabolism: it has been shown to control the expression of genes involved in oxidative phosphorylation, and the loss of ALDH1L1 expression has been shown to significantly affect acylcarnitine profiles [2,15,16,26]. Furthermore, the ratio of serine-to-glycine has been shown to alter the expression of genes involved in fatty acid metabolism, possibly through changing promoter methylation [27]. Our results suggest that the loss of ALDH1L1 disrupts the crosstalk between the serine/glycine metabolism and the mitochondrial fatty acid metabolism, which may lead to mitochondrial dysfunction.

The metabolism of pantothenate, recognized as vitamin B5, was another metabolic process that was correlated with serine and glycine in KO mice, but not in WT mice. Pantothenate has a pivotal role as the acyl group carrier, a function highly relevant to lipid metabolism. Specifically, pantothenate is involved in Coenzyme A (CoA) biosynthesis and as a prosthetic group in the Acyl Carrier Protein (ACP) of the FAS complex [28]. CoA, functioning as a ubiquitous cofactor, partakes in diverse metabolic reactions, including the synthesis and degradation of fatty acids, Tricarboxylic Acid (TCA) cycle operation, and phospholipid synthesis. CoA is imperative for fatty acid oxidation, pyruvate and α-ketoglutarate metabolism, sterol metabolism, and the acetylation of diverse molecules to modulate their transport characteristics or functionalities [29]. Pantothenate can be interconverted into different chemical forms within the cell. Intracellular CoA levels are the result of an equilibrium between these forms, with the assembly of CoA occurring in five enzymatic steps. Particularly, the phosphorylation of pantothenate to 4′phosphopantothenate catalyzed by pantothenate kinase constitutes the rate-limiting and highly regulated step in this biosynthetic pathway. ACP, derived from apo-ACP and CoA, assumes a pivotal role in fatty acid synthesis through activation of the acetyl–fatty acid synthase complex [29]. Our results suggest that, in the absence of ALDH1L1, glycine and serine are more actively involved in pantothenate and CoA biosynthesis pathway and this may be a mechanism for causing widespread metabolic reprogramming seen in ALDH1L1 loss. Curiously, similar to ACP, ALDH1L1 has the phosphopantetheine prosthetic group [30]. It is not clear at present whether this aspect would have an effect on pantothenate/CoA metabolism.

## 5. Conclusions

In conclusion, this study provides insights into the coupling of serine and glycine to other metabolic processes in the presence or absence of ALDH1L1 expression. Future targeted metabolomics and metabolite flux analyses are needed to validate the relevance of these findings to the ALDH1L1 function. Our current study shows that this analytic approach can be useful in identifying potential metabolite flux pathways as well as identifying biological functions of metabolites. This approach has the advantage of being label-free and does not require “a priori selection” of a substrate of interest. Future studies could explore the correlation of these pathways with ALDH1L1-related diseases, such as cancer, and assess their implications for developing novel treatments. Additionally, further investigations should delve into the impact of a folate-deficient diet and regulated diet on the correlation of glycine and serine to these metabolic pathways, potentially uncovering dietary interventions for the treatment of ALDH1L1-related diseases. In addition, future research could extend these findings to known polymorphisms in the *ALDH1L1* gene, which would allow a better understanding of how the function of ALDH1L1 varies across populations. Replicating the study’s outcomes in a human population has the potential to translate into precision medicine strategies tailored to individuals’ genetic information. Lastly, investigation into other amino acids using this correlation approach could be informative to see if these same pathways are coupled to other amino acids aside from serine and glycine. Overall, such studies deepen our understanding of the metabolic role of ALDH1L1, uncover unknown metabolic links, and highlight their implications for health and disease.

## Figures and Tables

**Table 1:** Liver metabolites with a correlation *p*-value *<* 0.05 compared to peak intensity of glycine in KO genotype mice.

Liver Metabolite	*p*-Value
Cystathionine	1.71 × 10^−5^
Serine	2.38 × 10^−5^
Tryptophan	0.00223
Valerylcarnitine	0.00287
Guanosine	0.00545
Glutamine	0.00701
5-Hydroxytryptophan	0.00792
Tryptamine	0.00892
Xanthine	0.0155
Histidine	0.0178
4-Hydroxy-L-Phenylglycine	0.0204
Octanoylcarnitine	0.0204
Cytidine	0.0239
5′-Deoxyadenosine	0.0297
Beta-Nicotinamide mononucleotide	0.0316
Tyrosine	0.0316
Sarcosine	0.0325
4-Guanidinobutyric acid	0.0325
1-Methyl-L-histidine	0.0335
Hexanoyl Glycine	0.0366
Oxoproline	0.0376
Aspartic acid	0.0387
Histidinol	0.0387
N-Methyl-glutamic acid	0.0410
Anserine	0.0458
Pantothenate	0.0496

**Table 2:** *p*-value summary of significantly correlated metabolic pathways with serine, glycine, or the serine-to-glycine ratio for both liver and plasma metabolites in WT and KO mice.

Metabolic Pathway	Glycine vs. Plasma		Serine vs. Plasma		Serine-to-Glycine Ratio vs. Plasma		Glycine vs. Liver		Serine vs. Liver		Serine-to-Glycine Ratio vs. Liver	
	WT	KO	WT	KO	WT	KO	WT	KO	WT	KO	WT	KO
Alanine, aspartate and glutamate metabolism						0.0042	0.026	0.0011				
Aminoacyl-tRNA biosynthesis			0.014	0.062	0.025	1.94 × 10^−4^	0.071	1.08 × 10^−4^	0.080	1.36 × 10^−7^		
Arginine and proline metabolism		0.097			0.092			0.026		0.038		7.36 × 10^−4^
Arginine biosynthesis	0.003					0.012		0.023	0.0079	0.0022		
beta-Alanine metabolism			0.081				0.015	0.0049		0.0075	2.22 × 10^−4^	0.0015
Biotin metabolism	0.058			0.083								
Butanoate metabolism	0.086											
Cysteine and methionine metabolism		0.085					0.036	0.018		0.026		
D-Glutamine and D-glutamate metabolism	0.035					0.002		0.099				
Galactose metabolism							0.025					0.038
Glutathione metabolism	0.011					0.045			0.030		0.008	9.66 × 10^−6^
Glycerolipid metabolism	0.092											
Glycerophospholipid metabolism												0.064
Glycine, serine and threonine metabolism					0.0090		0.0032	0.0023	0.0040	0.028	0.091	4.23 × 10^−4^
Glyoxylate and dicarboxylate metabolism						0.057						
Histidine metabolism	0.092		0.062		0.019		3.34 × 10^−4^	1.11 × 10^−4^	0.010	8.32 × 10^−6^	0.024	
Linoleic acid metabolism									0.049		0.075	0.057
Lysine degradation		0.065			0.043						0.056	
Nicotinate and nicotinamide metabolism			0.058					0.03	3.39 × 10^−4^	0.034		
Nitrogen metabolism	0.035					0.0021		0.10				
Pantothenate and CoA biosynthesis				0.011				0.041		0.053		
Phenylalanine metabolism										0.022		
Phenylalanine, tyrosine and tryptophan biosynthesis						0.048		0.067		0.002		
Purine metabolism						8.19 × 10^−4^			0.025	0.039	0.076	
Pyrimidine metabolism	0.021				0.096	0.011		0.028		0.040		
Riboflavin metabolism			0.016									
Sphingolipid metabolism					0.031							
Tryptophan metabolism			0.010	2.84 × 10^−4^	0.016			0.032		0.0078		
Tyrosine metabolism						0.092						
Valine, leucine and isoleucine biosynthesis				0.067								
Vitamin B6 metabolism		0.024										

*p*-values less than 0.1 are displayed in the table.

## Data Availability

Data are contained within the article or Supplementary Material.

## Supplementary Materials

The following supporting information can be downloaded at: https://www.mdpi.com/article/10.3390/metabo14120696/s1, Table S1: Correlation of all in-house matched metabolites with glycine, serine, and the serine-to-glycine ratio.
